# Blood volume measurement using cardiovascular magnetic resonance and ferumoxytol: preclinical validation

**DOI:** 10.1186/s12968-018-0486-3

**Published:** 2018-09-10

**Authors:** Rajiv Ramasawmy, Toby Rogers, Miguel A. Alcantar, Delaney R. McGuirt, Jaffar M. Khan, Peter Kellman, Hui Xue, Anthony Z. Faranesh, Adrienne E. Campbell-Washburn, Robert J. Lederman, Daniel A. Herzka

**Affiliations:** Division of Intramural Research, National Heart Lung and Blood Institute, National Institutes of Health, Building 10, Room 2C713, 10 Center Drive, Bethesda, MD 20892 USA

**Keywords:** Heart failure, CMR, MRI, Ferumoxytol, Blood volume, *T*_1_ mapping

## Abstract

**Background:**

The hallmark of heart failure is increased blood volume. Quantitative blood volume measures are not conveniently available and are not tested in heart failure management. We assess ferumoxytol, a marketed parenteral iron supplement having a long intravascular half-life, to measure the blood volume with cardiovascular magnetic resonance (CMR).

**Methods:**

Swine were administered 0.7 mg/kg ferumoxytol and blood pool *T*_1_ was measured repeatedly for an hour to characterize contrast agent extraction and subsequent effect on *V*_blood_ estimates. We compared CMR blood volume with a standard carbon monoxide rebreathing method. We then evaluated three abbreviated acquisition protocols for bias and precision.

**Results:**

Mean plasma volume estimated by ferumoxytol was 61.9 ± 4.3 ml/kg. After adjustment for hematocrit the resultant mean blood volume was 88.1 ± 9.4 ml/kg, which agreed with carbon monoxide measures (91.1 ± 18.9 ml/kg). Repeated measurements yielded a coefficient of variation of 6.9%, and Bland-Altman repeatability coefficient of 14%. The blood volume estimates with abbreviated protocols yielded small biases (mean differences between 0.01–0.06 L) and strong correlations (*r*^2^ between 0.97–0.99) to the reference values indicating clinical feasibility.

**Conclusions:**

In this swine model, ferumoxytol CMR accurately measures plasma volume, and with correction for hematocrit, blood volume. Abbreviated protocols can be added to diagnostic CMR examination for heart failure within 8 min.

## Background

The hallmark of heart failure is volume overload [1, 2]. Clinical evaluation includes qualitative auscultatory markers of cardiac wall stress (gallops) and lung water (rales), peptide biomarkers of wall stress (natriuretic peptides), qualitative radiographic markers of interstitial lung fluid (infiltrates and Kerley lines etc.), cardiac imaging and invasive markers of pressure overload. Initial management of decompensated heart failure chiefly consists of diuresis to reduce volume overload. Nevertheless, quantitative measures of blood volume are generally not employed clinically, because they are not conveniently accessible and because their value has not been tested.

Accurate measurement of blood volume, if readily accessible, might impact management of chronic and decompensated chronic heart failure [3]. Previous studies have characterized patients with heart failure to have a total blood volume of 20% above body-weight predicted values [4–6].

Current methods to measure blood volume include controlled inhalation of carbon monoxide to tag circulating hemoglobin, or tracer dilution using Evans blue dye or radiopharmaceuticals [4, 7–10], and are infrequently used clinically because of ionizing radiation, cost, and/or complexity in stand-alone examinations. Cardiovascular magnetic resonance (CMR) dilution measurement of plasma blood volume has been attempted in rats [11] using gadopentetate dimeglumine (Gd-DTPA), which has a relatively short distribution and elimination kinetics that limit measurement accuracy and feasibility. Gadofosveset trisodium, which reversibly binds plasma albumin, might be an attractive alternative because of its a longer intravascular half-life [12] except but it exhibits significant and rapid extravascular distribution, and has been withdrawn from marketing because of poor sales. Hence, we sought to develop a convenient gadolinium-free method to quantify blood volume by using an agent with longer half-life, ferumoxytol, so that it can possibly be included in a standard CMR examination.

Ferumoxytol is a superparamagnetic iron nanoparticle indicated for iron replacement in iron-deficiency anemia. It is an alternative to gadolinium-based CMR contrast agents, which are associated with nephrogenic systemic fibrosis in patients with renal disease, and which are under scrutiny because of unexpected cerebral gadolinium accumulation [13–15]. Ferumoxytol exhibits marked relaxivity effects [16–18], and is a candidate tracer for blood volume measurement because of its long intravascular half-life of 9–14 h and low extravascular biodistribution [19].

The aim of this study is to assess the in vivo feasibility and repeatability of measuring blood volume using ferumoxytol tracer dilution. The accuracy of this measurement is compared against a reference carbon monoxide hemoglobin binding technique. We characterize measurement stability, in face of ferumoxytol elimination from the blood compartment at reduced doses, over a period of 1 h. We also performed Monte Carlo simulations to minimize the number of *T*_1_ measurements and produce an abbreviated acquisition protocol.

## Methods

### Animals

All procedures were approved by the institutional Animal Care and Use Committee and performed according to NIH guidelines. Swine (*N* = 6 Yucatan, 35–50 kg, S&S Farms, USA) were pre-medicated with glucocorticoids and anti-histamine [20, 21]. Anesthesia was maintained with mechanical ventilation and inhaled isoflurane, and euvolemia restored after overnight fast using 10–15 ml/kg isotonic saline 20 min before blood volume measurements. Femoral arterial and venous femoral introducer sheaths were placed percutaneously.

### Ferumoxytol relaxivity in blood in vitro

CMR was performed at 1.5 T (Aera, Siemens Healthineers, Erlangen, Germany), using two standard body arrays. Longitudinal relaxivity *r*_1_ was characterized prior the repeatability study in a Yorkshire swine sample. A series of dilutions of ferumoxytol (0.10–1.72 mM) in 50 mL heparinized swine blood in a heated water bath (35–41 °C) were characterized with *T*_1_ measurements using with SAturation-recovery single-SHot Acquisition (SASHA) with 11 exponentially spaced saturation times ranging from 100 to 10,000 ms, and a simulated cardiac interval of 1 s (acquisition parameters below).

### Ferumoxytol blood volume measurement in vivo

Two successive imaging sessions tested the repeatability of the blood volume measurement at euvolemia. Based on a target post-contrast *T*_1_ of approximately 300 ms for optimal accuracy and precision in the *T*_1_ measurement [11], animals received 0.7 mg/kg (0.011 mM/kg) ferumoxytol diluted in 50 ml saline infused slowly ~ 0.4 ml/sec. As ferumoxytol elimination from the blood is faster at lower concentrations [19], each animal was imaged for an hour to characterize the resultant change in *T*_1_. Three animals underwent an additional imaging session at a reduced dose of ferumoxytol (*N* = 1 at 10%, 20% or 50% of the 0.7 mg/kg dose). Successive imaging sessions were 3–4 days apart. For each imaging session, total blood volume was compared using both CMR and carbon monoxide (CO) rebreathing techniques [22].

### Carbon monoxide blood volume measurement in vivo

A reference blood volume was measured using the absorption of CO through a rebreathing apparatus constructed according to the methods proposed by Schmidt et al. [22]. Three baseline samples were drawn from the femoral artery and analyzed for carboxyhemoglobin (COHb) using a blood-gas analyzer (Avoximeter 4000, Accriva Diagnostics, Werfen Company Bedford, Massachusetts, USA).

Following baseline measurements, a 1 ml/kg (max 50 ml) volume of CO was administered to the rebreathing apparatus, and the reservoir bag squeezed to simulate a single inhalation held for 10 s. The animals were then manually ventilated for 2 min before reverting to normal ventilation using a 3-way stopcock. Blood was sampled to measure COHb at 6, 7 and 8 min following CO administration.

CO rebreathing allows for the estimation of blood volume *V*_*blood*_ based on the determination of *V*_*CO*_, the volume of CO administered through rebreathing [22]:

1 $$ {V}_{blood}={V}_{CO}\frac{K\ast 100}{\left[ Hb\right]\ast \Delta  \%\left[ COHb\right]\ast {n}_H}, $$

where *K* is a Boyle’s Law volume correction factor for standard temperature and pressure, the concentration of hemoglobin is given by [*Hb*] in g/L, and the hemoglobin oxygen capacity is given by Hüfner’s number, *n*_*H*_=1.31 ml/g [23]. The change in the concentration of COHb, *∆* % [*COHb*], is calculated from the average baseline and the average over 6 to 8 min post-administration of CO.

To account for systematic losses in the total volume of carbon monoxide administered (*V*_*CO*_) after rebreathing, the following corrections were made. First, a 1% loss due to the affinity of CO to Hb was assumed. Second, corrections for the volume of CO not administered through rebreathing and the volume expired to the air following rebreathing were applied as detailed by Schmidt et al. [22]. The volume of CO remnant in the spirometer was estimated by the product of the rebreathing apparatus volume and the concentration of CO ([*CO*]) in the rebreathing bag as determined by a CO monitor (Dräger Pac 3500, Dräger, Lübeck, Germany). The mean measured rebreathing bag volume was 2.7 ± 0.3 L, the spirometer’s volume was measured to be 0.04 L, and the lung volume of the swine was assumed to be 1 L [24]. The volume of CO removed by the body was estimated from the average [*HbCO*] over 6–8 min, using a previous finding that the concentration of COHb is related to the expired CO (expired CO = 5.09[*COHb*] + 2.34 ppm [25]), and assuming an alveolar ventilation rate of 5 L/min [22], and the average of the blood sampling times (7 min) as the measurement time.

### Quantification of blood volume with ferumoxytol

The total circulating blood volume *V*_*blood*_ can be estimated by the plasma blood volume *V*_*plasma*_, using the change in longitudinal relaxation rate *R*_1_ = 1/*T*_1_ after administration of contrast agent (CA) in conjunction with hematocrit. The change in relaxation rate in response to the infusion of CA is given by:

2 $$ {R}_1(t)={R}_{1,\varnothing }+{r}_i\left[ CA\right] $$

where *R*_1, ∅_ represents the native longitudinal relaxation rate before contrast, *r*_1_ is the agent’s relaxivity (mM^− 1^ s^− 1^), and [*CA*] is the concentration in mM. *R*_1_(*t*) decreases towards *R*_1, ∅_ exponentially as the agent is removed from circulation. As iron concentration is given by [*CA*] = *n*_*Fe*_/*V*, the plasma volume estimate *V*^′^(*t*) can be calculated thus:

3 $$ {V}^{\prime }(t)=\frac{r_1{n}_{Fe}}{\Delta {R}_1(t)}, $$

where *∆R*_1_(*t*) = *R*_1_(*t*) − *R*_1, ∅_, and *n*_*Fe*_ is the amount of iron in millimoles. Though *n*_*Fe*_ is time-dependent as the ferumoxytol is extracted from circulation, it is treated as constant, as the changing concentration of CA is measured within *R*_1_(*t*). The injected amount of iron (*n*_*Fe*_) is given by the product of iron concentration of ferumoxytol (30 mg/ml), the molar mass of iron (55.845 g/mol) and the injected volume of ferumoxytol in ml. Sequential measurements of *R*_1_(*t*) can be used to calculate *V*^′^(*t*) using Eq. 3. As posited by Pannek et al. for Gd-based agents, *V*_*plasma*_ can then be calculated by fitting the estimated ‘time-dependent’ plasma volume, *V*^′^(*t*) [11], assuming an exponential elimination:

4 $$ {V}^{\prime }(t)={V}_{plasma}\exp \left(\raisebox{1ex}{$t$}\!\left/ \!\raisebox{-1ex}{$\tau $}\right.\right), $$

where *τ* is the time constant for extraction of ferumoxytol from circulation. As physiology is typically reported in terms of half-life, this term shall be reported, with the half-life *t*_1/2_ = *τ* × ln 2. For the purposes of log-linear regression, Eq. (4) can be expressed as follows:

5 $$ \log \left({V}^{\prime }(t)\right)=\log \left({V}_{plasma}\right)+t/\tau, $$

thus *V*_*plasma*_ can be determined from the y-intercept. The total blood volume *V*_*blood*_ can be calculated by normalizing *V*_*plasma*_ by the hematocrit *Hct*, the ratio of the volume of red blood cells *V*_*RBC*_ to *V*_*plasma*_:

6 $$ {V}_{blood}={V}_{plasma}+{V}_{RBC} $$

7 $$ {V}_{blood}=\frac{V_{plasma}}{1- Hct} $$

Hence, blood *T*_1_ measurements pre- and post-ferumoxytol can be used to estimate *V*_*blood*_.

### CMR protocol

All SASHA *T*_1_ measurements were acquired in 4-chamber orientation to maximize the number of blood pool pixels in both the left ventricle (LV) and right ventricle (RV) at end-diastole. To measure the coefficient of variation in *R*_1_, five *T*_1_ maps were acquired pre-contrast, and the average of these were taken for the baseline measurement of *R*_1, ∅_. Post-contrast *T*_1_ maps were subsequently acquired every 2 min up to 60 min, yielding ~ 28 *T*_1_ measurements. Breath-hold SASHA imaging parameters were: TE/TR 1.01/2.02 ms, FOV 360 × 270 cm; acquired resolution 1.88 × 2.50 mm, reconstructed resolution 1.88 × 1.88 mm, slice thickness 8 mm, acceleration factor 3 [26]. A customized SASHA protocol used 17 images for 2-parameter *T*_1_ fitting: an initial ‘equilibrium’ image with no saturation pulse, followed by saturation preparation and imaging in successive heart beats: 8 each with saturation times of 200 and 400 ms [27].

### Image analysis

Regions of interest (ROIs) were manually drawn within the LV and RV for all *T*_1_ maps (typically ~ 150/60 pixels for the LV/RV), and myocardial ROIs were drawn at baseline, 4, 20 and 60 min. Plasma volume and ferumoxytol half-life was fitted from 20 to 60 min using Eq. 5 and estimation of the coefficient of determination (*r*^2^) was performed in Matlab (Mathworks, Natick, Massachusetts, USA). *V*_*blood*_ was calculated following Eq. 7 and normalized by the animal weight to yield the final estimate in ml/kg.

### Abbreviated protocols

The reference acquisition for the estimation of blood volume acquired 26 measurements (5 pre- and 21 post-contrast) spanning over 60 min. We use this data, along with Monte Carlo simulations to develop 3 optimized abbreviated acquisition strategies differing in amount of data, time span, and accuracy. A single baseline *T*_1_ measurement was used for simulated and in vivo data.

- **Protocol #1** (1 post-contrast *T*_1_ measurement) represents the simplest scheme in which a single *T*_1_ measurement at 4 min post-contrast is used to estimate blood volume (similar to Pannek, et al. [11]).*V*_*blood*_ is determined directly from Eq. (3) after correction for hematocrit. Protocol #1 ignores for any time-dependence of the contrast washout and aims to acquire data at the earliest point post-administration, minimizing the overall time span of the measurement.
- **Protocol #2** (4+ post-contrast *T*_1_ measurements) acquires an increasing number of sequential *T*_*1*_ measurements starting with 20, 22 and 24 min after infusion. The additional data enables fitting, yielding for higher accuracy at the expense of time span.
- **Protocol #3** (4 post-contrast *T*_1_ measurements) used 3 sequential measurements (at 20, 22 and 24 min) anchored by a delayed measurement acquired between 30 and 60 min. Fitting is improved in accuracy by increasing the sampling time span, at the expense of workflow.

We also simulate a protocol with timing suitable for human subjects. The simulated ‘human’ protocols (3+ post-contrast *T*_1_ measurements) were composed of measurements every 2 min up to 20 min. This model assumed acute measurements would be possible in humans, given the expectation of tolerance to ferumoxytol.

To optimize the three acquisition strategies, Monte Carlo simulations were used. A model *T*_1_ recovery curve spanning 60 min post-contrast was created based on the observations from in vivo experiments (Table 1&2): *T*_1, ∅_=1600 ms, immediate post-contrast *T*_1_ = 230 ms, ferumoxytol half-life = 3.4 h, *v*_*Fe*_=1.0 ml, and *r*_1_ = 18 mM^− 1^ s^− 1^. Each simulation was performed 10,000 times with a *T*_1_ standard deviation of 0.6% (taken from the experimental coefficient of variation), the resultant curves were sub-sampled and fitted following Eq. (5). The standard deviation of plasma volumes $\left({\sigma}_{V_{plasma}}\right)$ and goodness-of-fit (*r*^*2*^) were recorded for each protocol.

**Table 1:** T_1_ values (mean ± SD, *n* = 12) at baseline and after administration of a 0.7 mg/kg dose of ferumoxytol, across the measurement protocol duration for the LV blood pool, RV blood pool and myocardial tissue (myo) from the repeatability study

	*T*_1_ Baseline (ms)	*T*_1_ Post-ferumoxytol (ms)		
		4 min	20 min	60 min
LV	1604.2 ± 27.7	232.0 ± 12.3	250.6 ± 15.7	281.4 ± 19.7
RV	1807.9 ± 96.8	235.9 ± 14.5	257.1 ± 20.3	289.0 ± 21.7
myo	1135.0 ± 80.2	837.2 ± 42.9	844.5 ± 63.3	860.1 ± 59.3

Three protocols were identified through Monte Carlo modelling, and compared to in vivo plasma volume measurements using correlation and Bland-Altman analysis.

### Statistical analysis

Coefficients of variation (CV) and repeatability coefficients (RC) were calculated for the differences in normalized blood volumes between the replicate euvolemic sessions, and for the baseline *T*_1_, using the following equations:

8 $$ CV=100\%\frac{\mu }{\sigma } $$

9 $$ RC=1.96\sqrt{\frac{\sum {\left({x}_i-\mu \right)}^2}{n-1}}\ \frac{100\%}{\mu } $$

where the data *x*_i_ composed of *n* measurements had mean *μ* and standard deviation *σ*. The accuracy of the CMR blood volume technique was compared to reference measurements using CO with a non-parametric paired test (Wilcoxon), in addition to a linear regression and Bland-Altman analysis. Low-dose half-lives and blood volumes were compared against the standard dose using Mann-Whitney U tests.

## Results

### In vivo summary

The baseline swine weight was 41 ± 6 kg (range 35–49 kg) and mean individual deviation from baseline was 0 ± 1 kg indicating no observable change in rapidly growing animals (*p* > 0.9, one-way ANOVA) over 19 days. Mean baseline heart rate was 118 ± 7 bpm, and baseline mean arterial pressure was 72 ± 14 mmHg. Ferumoxytol induced no serious adverse reactions and mean arterial pressure and heart rate variations were 4 ± 1 mmHg and 0 ± 1 bpm respectively, averaged over 10 min.

### Measurement of Relaxivity and T_1_

The relaxivity *r*_1_ of ferumoxytol in swine blood in vitro was 18.0 ± 0.4 mM^−1^·s^−1^. In vivo baseline *T*_1_ mapping yielded a CV of 0.58 ± 0.27% and 0.90 ± 0.63% for the LV and RV, respectively, and Bland-Altman repeatability coefficients of 1.0 ± 0.5% and 1.6 ± 1.1% respectively within the LV and RV.

**Fig. 1:**
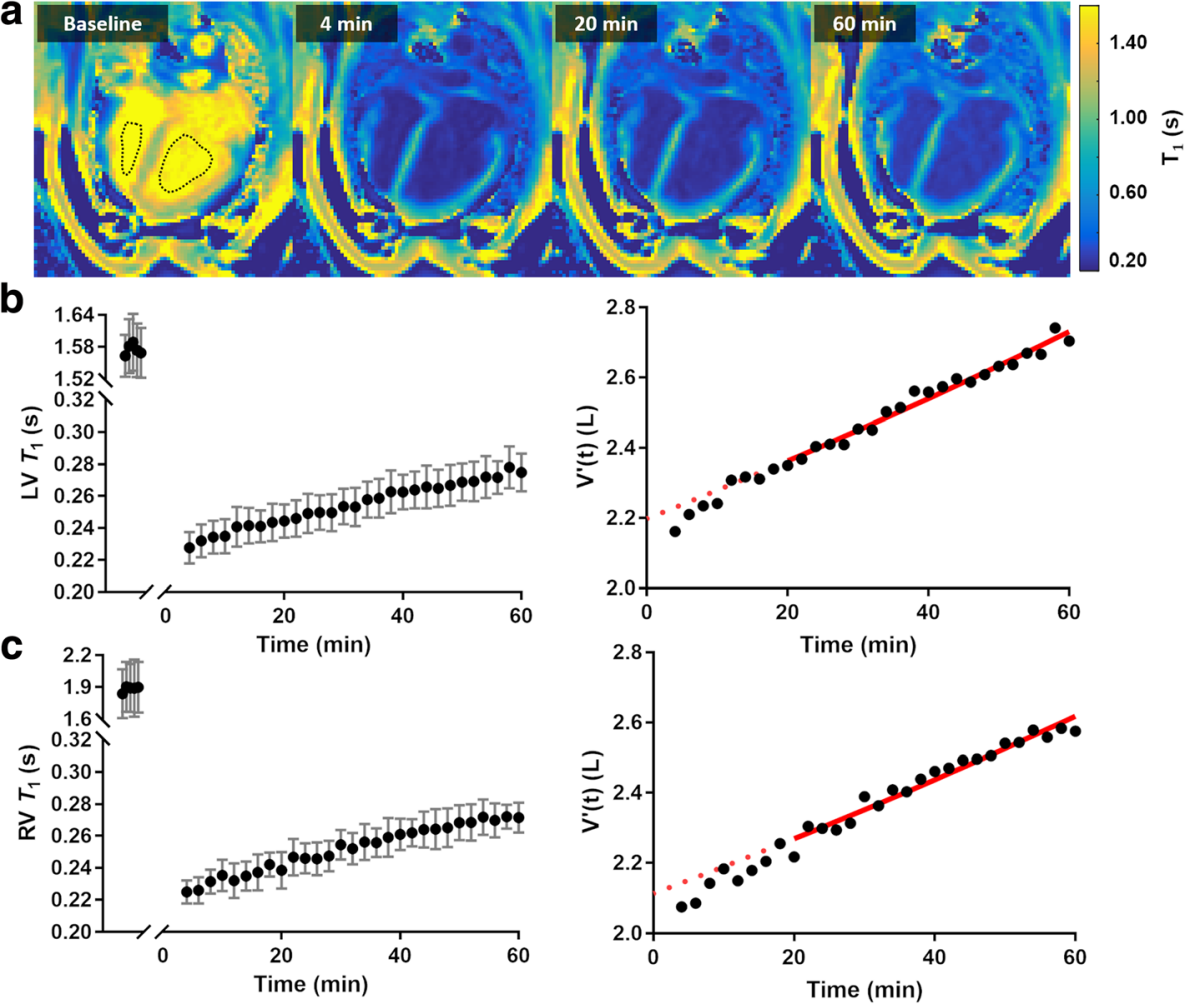
**a** Example long-axis *T*_1_ maps and regions-of-interest (ROI) over the duration of a study, and typical *T*_1_ (intra-ROI mean ± SD) recovery profile post-contrast and the corresponding volume *V*′(t), calculated from Eq. 3, for the (**b**) left-ventricle (LV) and paired (**c**) right-ventricle (RV). A linear fit from 20 to 40 min (solid line) is used to measure the half-life of the contrast agent. The plasma volume can be estimated by extrapolating to the model to 0 min (dotted line)

*T*_1_ maps before and after administration (at 4, 20 and 60 min) of ferumoxytol can be seen in Fig. 1a, and the partial recovery in blood pool *T*_1_ over the hour-long measurement period and the corresponding time-dependent volume estimation, as determined by Eq. 3, is shown for the LV and RV in Figs. 1b and c, respectively. A non-linear rate of contrast extraction was observed until ~ 20 min post-contrast administration, potentially due to a physiological response to ferumoxytol. Thus, fitting was constrained between 20 and 60 min (solid red line), and plasma volume estimated from the intercept (dotted line).

Estimates of blood volume were calculated separately from the LV and RV blood pools. Over the repeatability study, good adherence to the linear model was observed, with *r*^2^ = 0.97 ± 0.03 (mean ± SD) for the LV. Fitting was marginally weaker (*r*^*2*^ = 0.94 ± 0.10) for the RV, reflective of the larger intra-ROI variation. The percentage confidence intervals of plasma volume were 0.57% and 0.68% for the LV and RV, respectively. Bland-Altman analysis showed the RV non-significantly over-estimated plasma volume with a mean difference of 0.05 L (*p* > 0.27, paired t-test).

Table 1 shows the *T*_1_ values (mean ± SD) from the repeatability study for the LV and RV blood pools, and myocardium. The LV blood pool *T*_1_ decreased to 14.5% of baseline after a dose of 0.7 mg/kg ferumoxytol, and over the hour exhibited a 21.3% increase from the immediate post-contrast *T*_1_. Myocardial *T*_1_ reduced to 67% of the baseline and showed only a small amount of recovery at 60 min (2.7%).

### Blood volume: repeatability

**Fig. 2:**
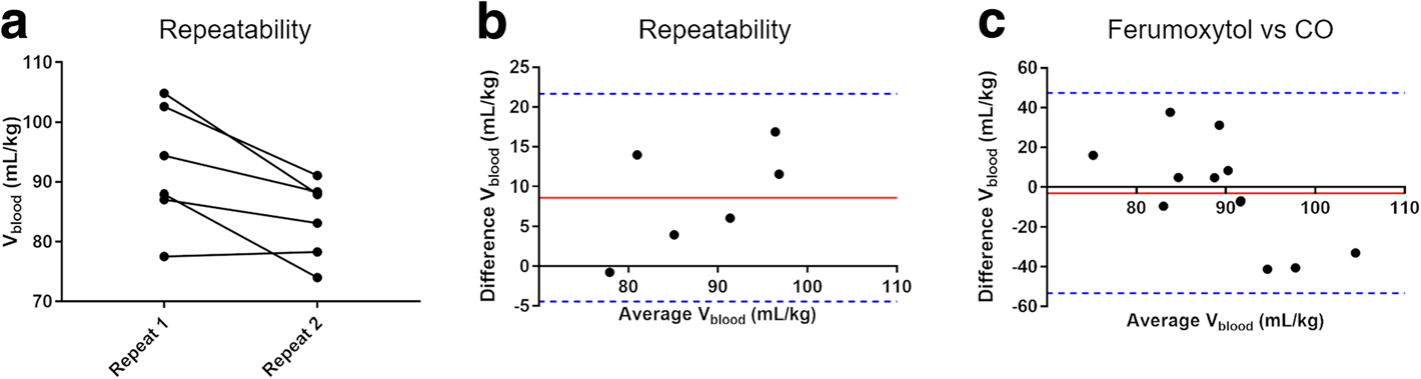
Repeatability of the blood volume (*V*_*blood*_) measurement: **a** Ladder plot between two repeat visits of *N* = 6 swine, **b** Bland-Altman comparison of technique reproducibility: mean difference and standard deviation = 8.6 ± 6.7 mL/kg. **c** Bland-Altman comparison to carbon monoxide estimates of circulating blood volume: mean difference and standard deviation = − 3.0 ± 25.7 mL/kg

Over the repeatability study, mean plasma volume (*V*_plasma_) was measured as 61.9 ± 4.3 ml/kg, mean blood volume (*V*_blood_) was measured as 88.1 ± 9.4 ml/kg using ferumoxytol. Blood hematocrit (*Hct*) was 29.5 ± 3.4% and Hb concentration 9.8 ± 1.1 g/dL. The repeatability of the normalized blood volume from two visits can be seen in Fig. 2a, with a coefficient of variation of 6.9%. The measurements exhibited a mean difference of 8.6 ml/kg (9.8%) between the studies, as can be seen in Fig. 2b. A potential magnitude-dependent bias was observed, and the repeatability coefficient of the technique was calculated to be 14%.

The CO reference measurement yielded a mean blood volume of 90.8 ± 18.9 ml/kg. There was no significant difference between blood volume estimated from ferumoxytol and CO (*p* = 0.67). Bland-Altman comparison (Fig. 2c) between the CMR and CO blood volume measurements yielded a small mean-difference (carbon monoxide was 2.7 ml/kg larger), though there is a potential trend in this data and a poor correlation *r*^2^ = 0.13. The measured CV for the CO blood volume was 18% and the Bland-Altman repeatability coefficient was 32%.

### Abbreviated protocols

**Fig. 3:**
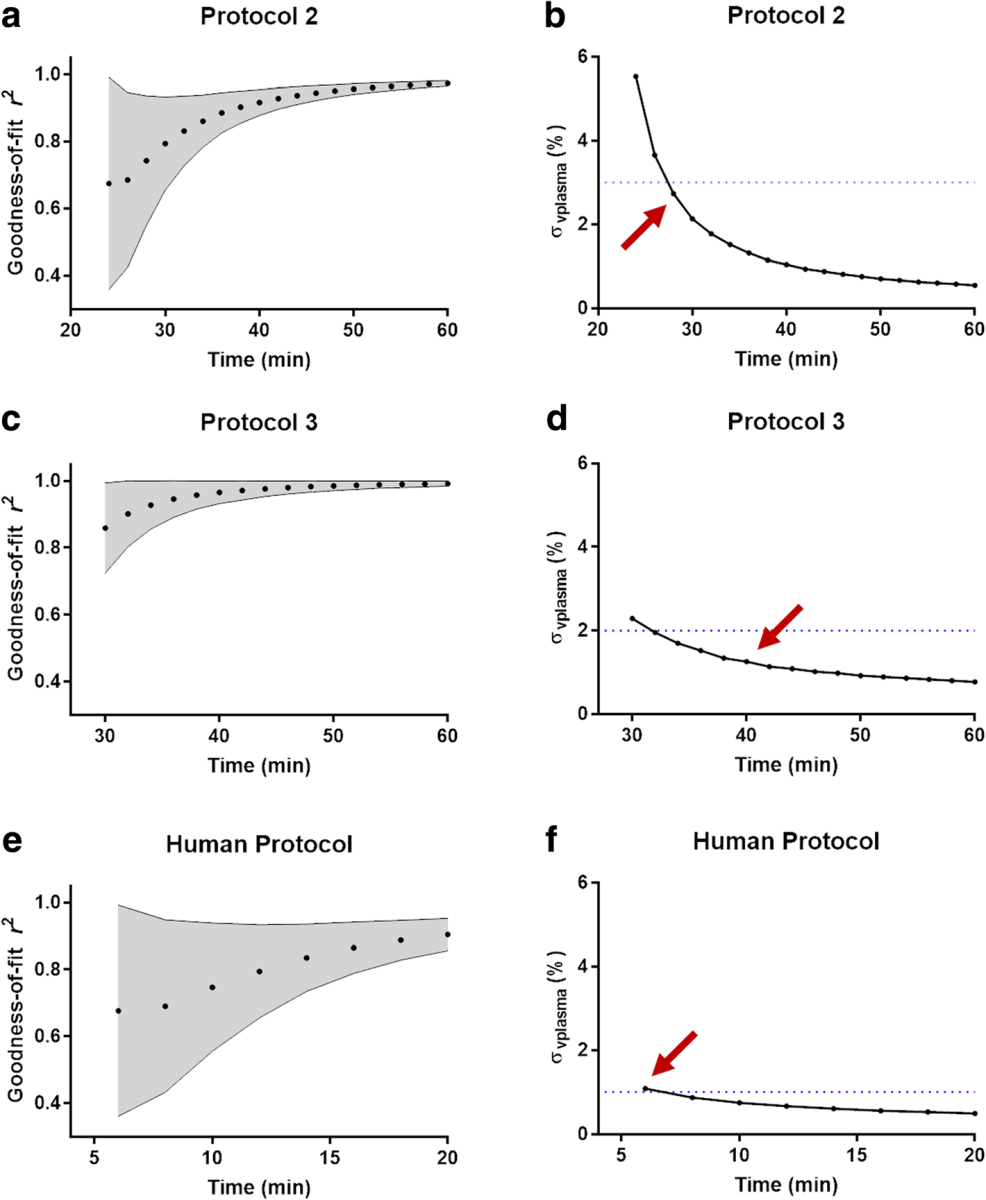
Results from Monte Carlo simulations (*n* = 10,000) on the effects on goodness-of-fit (*r*^2^) and plasma volume standard deviation (${\sigma}_{V_{plasma}}\Big)$ as a function of increasing the number of samples from 26 min (Protocol 2) (**a**, **b**) and increasing the time of the ‘anchor’ point from 30 min (Protocol 3) (**c**, **d**). Shaded regions correspond to the standard deviation of the goodness-of-fit (*r*^2^), and the points selected for in vivo analysis have been marked a red arrow. Dotted lines correspond to a 3, 2 and 1% plasma volume standard deviation in b, d and f respectively. Simulations of a potential ‘human protocol’ with an increasing number of acute post-contrast sampling (**e**, **f**) reveal that measurements as early as 6 min (red arrow) yield an approximate 1% standard deviation in plasma volume

Monte Carlo modelling of the full protocol yielded a goodness-of-fit *r*^2^ of 0.97, matching the in vivo results, and estimated plasma volume precision as 0.54%. Simulations predicted a more precise measurement of plasma volume with increasing experiment time (Fig. 3). Propagation of error analysis (on Eq. 3) indicates that a 4% error in the measurement of *T*_1_ (based on the observed intra-ROI standard deviation) would result in 4.7% error in the measurement of blood volume. For Protocol #2, though an increased number of acquisitions yielded improved plasma volume precision, a limit of 5 continuous measurements from 20 to 28 min was chosen for practicality, which resulted in error < 3% with a bias of 0 ml. Finally, for Protocol #3, an anchor measurement past 40 min resulted in error < 2%, with a bias of 0 ml. The proposed human protocol achieved ~ 1% error after measuring up to 6 min post-contrast (3 *T*_1_ measurements).

**Fig. 4:**
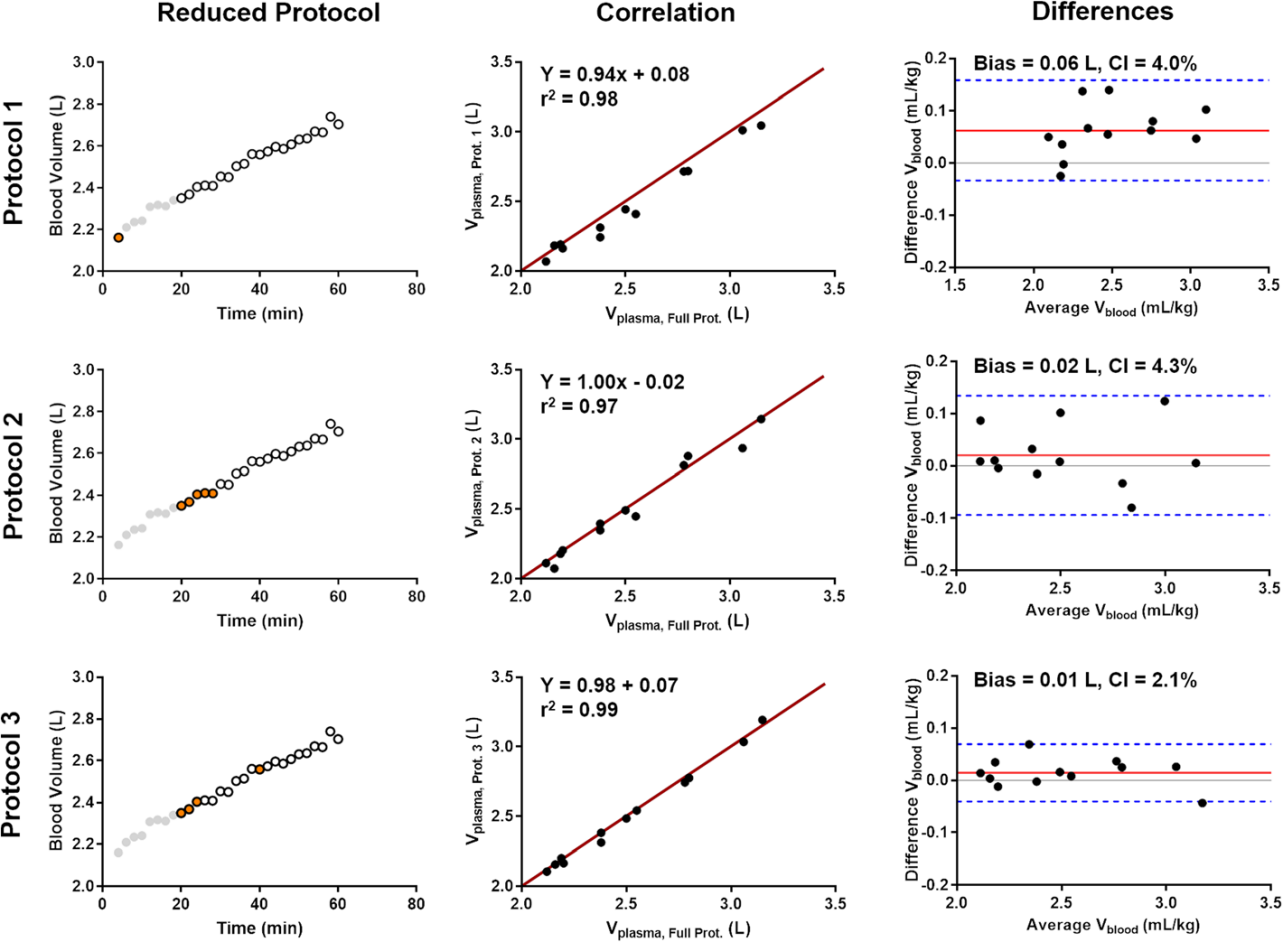
Comparison of optimal abbreviated acquisition protocols to fully-sampled reference 60 min acquisitions: linear correlations and Bland-Altman plots of biases for the repeated euvolemic conditions. The tested reduced-protocol acquisition points are represented by the orange circles in the left-hand column: Protocol 1 calculates plasma volume (*V*_plasma_) from a single point; the earliest post-contrast administration point. Protocol 2 and Protocol 3 respectively use an early set of successive measurements (5 points) and a reduced set of early measurements and a late ‘anchor’ (4 points) for optimal linear fitting, respectively. Each technique agrees well with the fully sampled dataset, though increased sampling over a longer period improves both bias and correlation

Figure 4 shows the comparison of the three abbreviated protocols based on the simulated precision in blood volume. The simplest scheme - Protocol #1 yielded an *r*^2^ of 0.98 and a Bland-Altman mean under-estimation of 0.06 L (2.4%). Protocol #2, with 5 successive measurements, yielded *r*^*2*^ = 0.97 and a Bland-Altman mean under-estimation of 0.02 L (0.8%). Protocol #3 with 3 early measurements followed by an ‘anchor’ at 40 min produced an r^2^ = 0.99 and a Bland-Altman mean under-estimation of 0.01 L (0.4%). These results suggest that all the abbreviated protocols can accurately calculate plasma volume.

### Reduced dose ferumoxytol

The effects of a reduced ferumoxytol dose on the normalized change in LV *T*_1_ from baseline, half-life estimated blood volume, and percentage difference from the blood volume measured in the repeatability study, can all be seen in Table 2. An expected decrease in Δ*T*_1_ was observed with smaller doses, and a significantly shorter half-life was measured across the reduced doses compared to the 0.7 mg/kg dose (*p* < 0.02, Mann-Whitney U). No significant differences in blood volume were calculated using the reduced doses (*p* = 0.95, Mann-Whitney U). Aside from the 50% dose, the estimated blood volumes were within the variability calculated by the repeatability coefficient.

**Table 2:** Comparison of measuring blood volume (*V*_blood_) with reduced ferumoxytol doses (*N* = 1, for each dose) in mg/kg of iron (Fe), and percentage of the typical dose used in this study (*N* = 12). Smaller doses resulted in a reduced change in post-contrast LV *T*_1_, and a shorter ferumoxytol half-life, but did not produce a significantly different normalized blood volume (*p* = 0.95, Mann-Whitney U), and except for the 50% dose, the percentage differences from the blood volumes for each subject were within the calculated repeatability coefficient (14%)

Conc. Fe (mg/kg)	Δ*T*_1_ (%)	Half-life (hr)	*V*_blood_ (mL/kg)	%Δ*V*_blood_
0.69 ± 0.01	−85.5 ± 0.7	3.43 ± 0.49	88.1 ± 9.4	–
0.30 (50%)	− 74.6	2.55	79.9	−17.5
0.23 (20%)	− 65.9	2.93	95.4	12.1
0.13 (10%)	−53.9	2.51	86.2	−5.7

## Discussion

We describe a CMR technique to measure total circulating blood volume using injected ferumoxytol and compared it with reference standard carbon monoxide inhalation in swine. We optimized the measurement strategy to propose clinical protocols requiring a maximum of 2–6 additional *T*_1_ measurements. The technique avoids frequent blood tracer sampling, avoids radioactive or dye tracers, and uses a small dose of parenteral iron supplement that can be used for first-pass and steady state CMR angiography.

### Measurement of blood volume

We found mean blood volume of 88.1 ± 9.4 ml/kg and 91.1 ± 18.9 ml/kg using ferumoxytol and carbon monoxide respectively. From Monte Carlo modelling, the calculated error in plasma volume was estimated to be 0.54% for the 60-min measurement, which suggests the method is precise. However, the values measured in this study are higher than reported blood volume values of swine (between 35 and 50 kg) range from 56 to 69 ml/kg in Yucatan swine (no weight range given) [28], 61–77 ml/kg in Chester-White [29], in 74–96 ml/kg in Duroc-Jersey swine [30], and between 56 and 68 ml/kg in Hampshire swine [31]. This variability may be in part to age and weights, it has been generally observed that older and larger swine will have a reduced normalized blood volume [31].

Plasma volume was measured using Eq. 3 for both the RV and LV blood pools. Though the baseline RV *T*_1_ is higher than LV (13%), both blood pools drop to approximately the same post-contrast *T*_1_ (2% RV/LV ratio). However, the mean difference of blood volume produced a (non-significant) 2% over-estimation, which would suggest that the post-contrast measurement is the dominant factor in volume quantification, and thus a single pre-contrast measurement is sufficient. This is confirmed by propagation of error analysis applied to Eq. 3, which indicated that a 1% error in *T*_1,0_ and *T*_1_ result in 0.17% and 1.81% errors in blood volume, respectively (data not shown). As the LV ROI had a higher number of pixels and yielded a smaller intra-ROI variation, this was chosen for the repeatability analysis. Under conditions of hyperoxia, blood hemoglobin *T*_1_ has been reported to increase [32], which is likely to be the source of left-right difference observed in this study at baseline.

In this study, the most accurate measurement of plasma volume required temporal monitoring of blood *T*_1_ as ferumoxytol was extracted from circulation to estimate the agent’s half-life through fitting. Since ferumoxytol has been reported to have a dose-dependent extraction rate (ranging from 9 to 14 h in humans) [19], assuming a half-life could lead to errors in the estimation of blood volume. In this study, we found a comparatively short mean half-life of 3.4 ± 0.5 h, potentially due to differences in swine physiology. We measured no significant differences in blood volume at lower doses, but further work is necessary to determine the minimal dose required in humans to achieve sufficient blood volume accuracy.

The *V*_plasma_ quantification is proportional to the relaxivity of ferumoxytol, *r*_1_. Others report *r*_1_ to be 19.0 ± 1.7 mM^− 1^ s^− 1^ at 1.5 T [16], in good agreement with our 18.0 ± 0.3 mM^− 1^ s^− 1^ in blood. Knobloch et al. reported a non-linear behavior of relaxivity in blood, particularly at higher concentrations (> 1 mM), which we avoided to minimize the effect of *T*_2_* dephasing. As ferumoxytol relaxivity is dependent on field strength, to achieve the same Δ*T*_1_, a higher dose of ferumoxytol would be required at 3 T [16]. Further investigation is required to characterize the accuracy of the blood volume measurements with low doses (< 1 mg/kg) of Ferumoxytol, as some variability was measured here. Additional measurements are required to see any effects on sensitivity at higher, more clinical doses (2–6 mg/kg), particularly due to non-linear effects on relaxivity at higher concentrations.

### Sources of error

The repeatability coefficient of porcine blood volume measured by ferumoxytol was 14%, which is higher than previous values of 8% using a ^131^I tracer [4]. Small inaccuracies in the ferumoxytol-based estimate may be introduced from error in the administered volume and *T*_1_ variation due to heart rate changes over the imaging session.

The calculated Bland-Altman repeatability coefficient of the CO technique (32%) was higher here than previous implementations 0.8–2.7% [8, 33, 34]. The potential sources of error in measuring blood volume with CO have been described [8, 35], but in our lab additional sources of error could include manually rebreathing anesthetized animals, systematic differences in measuring porcine COHb on a human blood-gas analyzer [36], and variability in the CO detector’s measurements of the remnant volume in the spirometer (coefficient of variation 25.3 ± 10.2%).

### Abbreviated protocols for clinical implementation

Abbreviated protocols yielded small biases (between 0.4–2.4%) and strong correlations (*r*^2^ = 0.97–0.99) to the reference 60 min experiment. The chosen abbreviated protocols yielded plasma volume repeatability coefficients of 9%, 14%, and 12% for protocols 1, 2 and 3, respectively.

Each protocol has advantages and disadvantages depending on the time constraints of a CMR exam. The most accurate and precise technique, Protocol #3, requires a ‘late’ measurement at 40 min post-contrast to improve the fit. This measurement improves as this acquisition moves later in time, but a prolonged measurement becomes unfeasible. The single post-contrast measurement, Protocol #1, is the fastest measurement, but is dependent on an immediate acquisition, and having a very accurate and precise *T*_1_ measurement. A short cluster of five measurements, Protocol #2, combine speed and the accuracy of longitudinal fitting. Protocols #2&3 are not dependent on two-minute interval acquisitions, thus by alternating a post-contrast *T*_1_ acquisition with standard diagnostic scans, these methods can be integrated in to a CMR workflow and could achieve higher accuracy and precision by broadening the measurement period.

The additional imaging time required to measure blood volume with these protocols are minor: one baseline and 1–5 post-contrast *T*_1_ maps. We anticipate that with short, breath-held *T*_1_ scans (~ 11 s), any of the abbreviated protocols, including the contrast administration, will require no more than 5 min of additional time to a CMR exam. A free-breathing *T*_1_ mapping technique (e.g. [37]) may be preferable to avoid repeated breath-held acquisitions, though each may take up to 60 s, and free-breathing techniques may yield an increase in *T*_1_ reproducibility.

Protocol 2 was optimized starting from 20 min given the non-linear acute behavior observed in swine, but we anticipate ferumoxytol will be better tolerated in humans, therefore *T*_1_ measurements could begin earlier. Modelling of this acute ‘human’ protocol (Fig. 3e-f) predicts that an accurate (0 L bias) and precise (~ 1% error) blood volume measurement could be achieved within a total experiment time (baseline, injection and post-contrast) of 8 min. In addition, as clinical dose of Ferumoxytol yield longer half-lives, a protocol with a few repeats post-contrast may also be feasible which do not require temporal fitting – however, further work is required to validate this.

### Limitations

Ferumoxytol, in higher iron replacement doses, has been associated with reported 0.2% incidence of anaphylactoid reactions and carries a black box warning from the US Food and Drug Administration [38]. The incidence and seriousness appear to be dose- and infusion-rate-dependent, and is probably lower in clinical practice [39–42].

We found Yorkshire and Sinclair mini swine breeds did not tolerate ferumoxytol (data not shown), but that with glucocorticoid and antihistamine premedication and prolonged 2-min infusion, Yucatan mini-swine tolerated ferumoxytol. We studied only healthy swine and not animals exhibiting neurohormonal perturbations characteristic of heart failure.

Finally, the reference CO measurement had much larger variability than previously reported in humans. Though the mean blood volume estimates agreed reasonably with the CMR measurements, there was a low correlation between these values, and therefore difficult to isolate animal variability from the repeatability of each technique.

## Conclusion

Ferumoxytol CMR can be used to measure plasma and total blood volume in vivo in swine, with comparable precision and accuracy to reference standard CO rebreathing methods. We design abbreviated acquisition protocols using Monte Carlo simulations and validate in vivo relative to a reference examination. These shorter acquisitions could provide estimates of blood volume in 8 min. Though the clinical value of direct blood volume measurements remains to be established, it may prove useful in the evaluation and management of heart failure and volume overload states as part of standard ferumoxytol-enhanced CMR.

## Abbreviations

CA: Contrast agent
CMR: Cardiovascular magnetic resonance
CO: Carbon monoxide
COHb: Carboxyhemoglobin
CV: Coefficient of variation
Fe: Iron
Hb: Hemoglobin
Hct: Hematocrit
LV: Left ventricle/left ventricular
RC: Bland-Altman repeatability coefficient
ROI: Region of interest
RV: Right ventricle/right ventricular
SASHA: SAturation-recovery single-SHot Acquisition
SD: Standard deviation
V_blood_: Total blood volume
V_plasma_: Plasma blood volume
